# Erythropoiesis-Stimulating Agents and Development of Cancer Among Patients Receiving Dialysis

**DOI:** 10.1001/jamanetworkopen.2026.0140

**Published:** 2026-02-27

**Authors:** Jae Young Kim, Jae Kwang Lee, Tae Ik Chang, Hyung Woo Kim

**Affiliations:** 1 Department of Internal Medicine, National Health Insurance Service Ilsan Hospital, Goyang-si, Republic of Korea; 2 Department of Internal Medicine, Institute of Kidney Disease Research, Yonsei University College of Medicine, Seoul, Republic of Korea; 3 Research and Analysis Team, National Health Insurance Service Ilsan Hospital, Goyang-si, Republic of Korea; 4 Yonsei Institute for Digital Healthcare, Yonsei University, Seoul, Republic of Korea

## Abstract

**Importance:**

Although erythropoiesis-stimulating agents (ESAs) are widely used to treat anemia in patients with kidney failure, concerns have been raised about their potential to promote tumor growth. However, the association between ESA use and incidence of cancer in patients undergoing long-term dialysis remains unclear.

**Objective:**

To examine the association between ESA use and cancer development among patients receiving long-term dialysis.

**Design, Setting, and Participants:**

This nested case-control study identified patients in the Korean National Health Insurance Service database with kidney failure who began long-term dialysis treatment and received ESAs between 2006 and 2017 in Korea. Each case with incident cancer was matched with 4 controls based on age, sex, follow-up time, year of dialysis initiation, and dialysis modality. Data were analyzed between August 2024 and August 2025.

**Exposure:**

ESA exposure was categorized as high or low dose according to the median of the mean weekly dose of each ESA drug.

**Main Outcomes and Measures:**

The primary outcome was newly diagnosed cancer occurring 6 months after the initiation of long-term dialysis. Conditional logistic regression, adjusted for potential confounders, was used to assess the association between ESA use and cancer development.

**Results:**

A total of 9776 patients (mean [SD] age, 62.2 [12.0] years; 6296 [64.4%] male) undergoing dialysis were included; 2320 patients with incident cancer were matched to 7456 controls. After multivariable adjustment, high-dose ESA use was found to be associated with increased odds of cancer development (adjusted odds ratio, 1.23; 95% CI, 1.11-1.35) compared with low-dose use. Stratified by age, the odds of cancer development were 0.90 (95% CI, 0.77-1.05) among patients aged younger than 60 years and 1.47 (95% CI, 1.30-1.67) among those aged 60 years or older.

**Conclusions and Relevance:**

In this case-control study of patients undergoing dialysis, high-dose ESA use was associated with greater odds of new cancer development. These results suggest that caution should be exercised to avoid aiming for excessively high hemoglobin levels during ESA therapy.

## Key Points

**Question:**

Is there an association between erythropoiesis-stimulating agents (ESAs) in the management of anemia and cancer risk in patients with kidney failure?

**Findings:**

In this case-control study of 9776 patients undergoing dialysis, high-dose ESA use was associated with higher odds of new cancer development among older patients (aged ≥60 years).

**Meaning:**

These findings suggest caution should be exercised to avoid aiming for excessively high hemoglobin levels during ESA therapy.

## Teaser

This case-control study evaluates the association between treatment with erythropoiesis-stimulating agents and cancer risk in patients receiving long-term dialysis.

## Introduction

Anemia affects more than half of patients with kidney failure, and its severity is linked to increased mortality, cardiovascular risk, and hospitalization rates.^1,2,3,4^ Recombinant erythropoiesis-stimulating agents (ESAs) have been the cornerstone of treatment of anemia in patients with kidney failure, as they can improve deficient endogenous erythropoietin production, reduce the need for transfusions, and improve quality of life. According to The 2012 Kidney Disease: Improving Global Outcomes Work Group,^5^ anemia guideline recommends initiating ESA therapy when hemoglobin levels fall below 10 g/dL (to convert hemoglobin to grams per liter, multiply by 10.0) and advise conservative titration. These recommendations reflect the recognition that overcorrection of anemia can increase the number of thromboembolic events and cardiovascular mortality and reduce overall survival.^6,7^

Mechanistic studies conducted to identify the underlying causes of the disadvantages associated with ESA use have revealed that ESAs can induce the activation of erythropoietin receptors, although the clinical evidence for increased angiogenesis and tumor growth remains controversial and subject to debate due to low-quality evidence in existing literature.^8,9,10^ Furthermore, large-scale evidence of cancer outcomes among patients with kidney failure remains sparse and inconsistent. However, large-scale evidence of cancer outcomes among patients with kidney failure remains sparse and inconsistent. An earlier study^11^ reported a higher risk of cancer-related mortality in patients with chronic kidney disease and a history of cancer using darbepoetin alfa compared with the placebo group. In contrast, another retrospective study^12^ found no association between voluntarily reported de novo cancer diagnoses and ESA use. Given the higher cancer burden among patients with kidney disease^13,14^ and ongoing safety concerns surrounding the routine use of ESAs, clarifying the association between ESA use and cancer risk in kidney failure is clinically important.

Therefore, this study aimed to investigate the association between ESA use and cancer development in patients with incident kidney failure undergoing dialysis using a nationwide population-based cohort from the Korean National Health Insurance Service (NHIS) database.

## Methods

### Data Source and Study Population

The entire population of patients aged 19 years or older with newly diagnosed kidney failure and without a prior kidney transplant who began and received long-term dialysis in Korea between January 1, 2006, and December 31, 2017, and received at least 1 ESA prescription was identified using the Korean NHIS database. The NHIS provides compulsory single-payer health insurance coverage for the entire Korean population, centralizing all inpatient and outpatient medical records.^15,16^ Kidney failure was defined as receipt of long-term dialysis or a kidney transplant, identified by specific insurance codes (called V codes) or dialysis-related intervention codes.^17^ The NHIS provides special insurance benefits to patients with kidney failure who require long-term dialysis or receive a kidney transplant. Once assigned, the kidney failure–specific code (eg, V001 for hemodialysis, V003 for peritoneal dialysis, and V005 for kidney transplant) is retained in all subsequent medical records and claims. The baseline date was defined as the date of issuance of the first kidney failure–related code. Patients were excluded if they had fewer than 180 days of dialysis after initiation (n = 3441) or a history of any cancer before the baseline date (n = 6806). Eligible patients were followed up from baseline to the time of kidney transplant, cancer diagnosis, death, or end of the study period (December 31, 2021). This study adhered to the Declaration of Helsinki and was approved by the institutional review board of NHIS Ilsan Hospital. Informed consent was waived owing to the use of deidentified data. The study followed the Strengthening the Reporting of Observational Studies in Epidemiology (STROBE) reporting guideline for case-control studies.

### Exposures and Covariates

The primary exposure was the mean weekly ESA dose, including epoetin alfa (short acting) and darbepoetin alfa or methoxy polyethylene glycol–epoetin beta (long acting). Patients with outpatient ESA prescriptions of 180 days or longer without a major class switch were considered as having been treated with ESAs. For patients treated with more than 1 type of ESA, the type that accounted for more than 90% of all prescriptions was considered the treatment medication. Patients treated with ESAs were divided into high-dose and low-dose groups according to the median of the mean weekly dose of each ESA drug, with patients (<0.1%) receiving the exact median dose assigned to the low-dose group.

Baseline characteristics included demographics (age, sex, residential area, and income level), comorbidities defined by inpatient or outpatient diagnosis codes (diabetes, hypertension, dyslipidemia, myocardial infarction, heart failure, peripheral vascular disease, cerebrovascular disease, dementia, chronic pulmonary disease, connective tissue disease, peptic ulcer disease, liver disease, hemiplegia, and AIDS), and health care facilities (primary care clinics and hospitals, including general hospitals and tertiary hospitals). Comorbidities were defined as the presence of at least 2 *International Statistical Classification of Diseases and Related Health Problems, Tenth Revision (ICD-10)* codes within 2 years before baseline. History of diabetes, hypertension, and dyslipidemia was further verified through medication records and *ICD-10* codes. The relevant codes for each comorbidity are listed in eTable 1 in Supplement 1. Health care facility level was defined as the facility where ESA prescriptions were issued in the highest proportion during the total treatment period.

### Selection of Cases and Controls

To identify case patients, the primary outcome was set as newly diagnosed cancer occurring 6 months after the initiation of long-term dialysis. A diagnosis of cancer was confirmed when both relevant *ICD-10* codes and cancer-specific insurance claim codes (V193 code) were present, ensuring eligibility for national financial support. The date of cancer onset was defined as the first date of a cancer-related diagnosis code in the claims data, and only the first cancer diagnosis after dialysis initiation was included as the outcome. Patients diagnosed with cancer at multiple sites of the body on the same date were included in each site-specific analysis. The relevant codes for each outcome are listed in eTable 1 in Supplement 1. We identified patients who developed the primary outcome between January 2006 and December 2021. Each patient was matched to 4 controls according to birth year, sex, follow-up time, year of dialysis initiation, and dialysis modality (hemodialysis or peritoneal dialysis) using incidence density sampling.^18^ Cases without matched controls at a ratio of 1:3 or 1:2 were excluded (Figure). Balance diagnostics are provided in the eFigure in Supplement 1.

**Figure.**
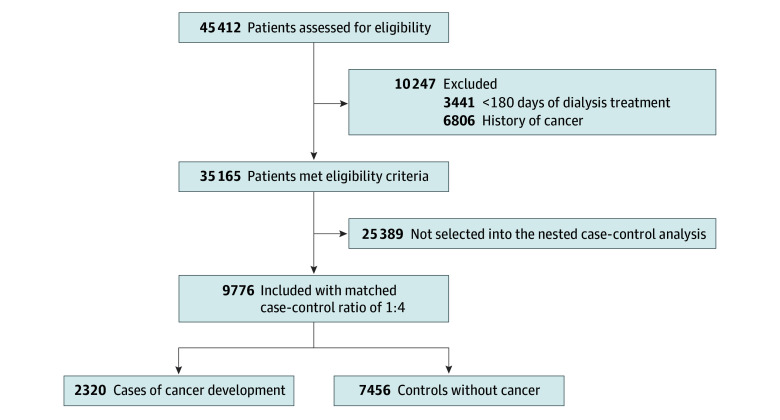
Flowchart of Study Cohort Counts in the nested case-control analysis are presented as unique individuals. In risk-set sampling, an individual may serve as a control prior to becoming a case.

### Statistical Analysis

Data from descriptive analyses are summarized as means (SDs) or numbers (proportions), as appropriate. For the primary analysis, conditional logistic regression was used to evaluate the association between ESA use and cancer development. In addition to the matching variables inherent to the nested case-control design, model 1 was adjusted for demographic variables (residential area and income level) and health care facility type. Model 2 was further adjusted for comorbidities (diabetes, hypertension, dyslipidemia, myocardial infarction, heart failure, peripheral vascular disease, cerebrovascular disease, dementia, chronic pulmonary disease, connective tissue disease, peptic ulcer disease, liver disease, hemiplegia, and AIDS) in addition to the variables in model 1. The findings for de novo cancer were expressed as adjusted odds ratios (AORs) with 95% CIs. Individual associations with frequent site-specific cancers (ie, ≥50 cases during the study period) were also examined. To assess the robustness of our findings, a sensitivity analysis was conducted considering only ESA use up to 6 months before the end of follow-up, based on the hypothesis that ESA requirements within 6 months of a cancer diagnosis may be a consequence of malignant neoplasm rather than a causal factor.^19,20^ Additionally, matched subgroups were analyzed based on age (<60 vs ≥60 years), sex, dialysis modality (hemodialysis vs peritoneal dialysis), ESA type (short vs long acting), health care facility type, and presence or absence of diabetes and hypertension to determine any differences potentially attributable to subgroup factors. All statistical analyses were performed using SAS version 9.4 (SAS Institute, Inc.) and Stata version 18 (StataCorp) from August 2024 to August 2025, with a significance level of *P* < .05; all *P* values were 2 sided.

## Results

### Baseline Characteristics of the Study Population

Of the 45 412 patients identified in the Korean NHIS database receiving long-term dialysis without a history of cancer who were treated with ESAs, a total of 9776 patients were included; 2320 case patients who developed cancers were matched to 7456 controls. The median follow-up was 6.5 years (IQR, 4.8–9.2 years). The mean (SD) age of participants was 62.2 (12.0) years, 64.4% were male and 35.6% female. In comparing differences in baseline characteristics, demographic and comorbidity profiles were relatively evenly distributed between the 2 groups. However, compared with the control group, the case group underwent dialysis more often at hospitals than at primary care clinics and had a lower likelihood of having previous peripheral vascular disease, a higher likelihood of having liver disease, and more frequent use of long-acting ESA medications (Table 1).

**Table 1.** Baseline Characteristics of Cases and Matched Controls

Characteristic	No. (%)			Crude OR (95% CI)
	Overall (N = 9776)	Cases (n = 2320)	Controls (n = 7456)	
Age, mean (SD), y^a^	62.2 (12.0)	62.4 (12.0)	62.1 (12.1)	NA
Sex				
Female	3480 (35.6)	784 (33.8)	2696 (36.2)	NA
Male^a^	6296 (64.4)	1536 (66.2)	4760 (63.8)	NA
Residential area				
Metropolitan	3206 (32.8)	756 (32.6)	2450 (32.9)	0.99 (0.94-1.05)
Large city	2338 (23.9)	574 (24.7)	1764 (23.7)	
Small city or rural area	4232 (43.3)	990 (42.7)	3242 (43.5)	
Income quartile				
First (lowest)	2866 (29.3)	685 (29.5)	2181 (29.3)	1.00 (0.95-1.03)
Second	1786 (18.3)	411 (17.7)	1375 (18.4)	
Third	2196 (22.5)	534 (23.0)	1662 (22.3)	
Fourth (highest)	2928 (30.0)	690 (29.7)	2238 (30.0)	
Health care facility				
Hospital	854 (8.7)	238 (10.3)	616 (8.3)	0.83 (0.70-0.98)
Primary care clinic	8922 (91.3)	2082 (89.7)	6840 (91.7)	
Dialysis modality^a^				
Hemodialysis	9300 (95.1)	2180 (94.0)	7120 (95.5)	NA
Peritoneal dialysis	476 (4.9)	140 (6.0)	336 (4.5)	NA
Comorbidity				
Diabetes	6093 (62.3)	1422 (61.3)	4671 (62.6)	0.95 (0.86-1.05)
Hypertension	9507 (97.2)	2250 (97.0)	7257 (97.3)	0.85 (0.59-1.21)
Dyslipidemia	6552 (67.0)	1527 (65.8)	5025 (67.4)	0.94 (0.85-1.05)
Myocardial infarction	575 (5.9)	114 (4.9)	461 (6.2)	0.81 (0.65-1.00)
Heart failure	1852 (18.9)	425 (18.3)	1427 (19.1)	0.96 (0.85-1.08)
Peripheral vascular disease	2204 (22.5)	469 (20.2)	1735 (23.3)	0.85 (0.75-0.95)
Cerebrovascular disease	1636 (16.7)	368 (15.9)	1268 (17.0)	0.90 (0.79-1.02)
Dementia	416 (4.3)	86 (3.7)	330 (4.4)	0.80 (0.62-1.03)
Chronic pulmonary disease	2416 (24.7)	582 (25.1)	1834 (24.6)	1.02 (0.91-1.14)
Connective tissue disease	638 (6.5)	170 (7.3)	468 (6.3)	1.18 (0.98-1.42)
Peptic ulcer disease	3394 (34.7)	768 (33.1)	2626 (35.2)	0.90 (0.82-1.00)
Liver disease	1260 (12.9)	378 (16.3)	882 (11.8)	1.42 (1.24-1.63)
Hemiplegia	257 (2.6)	58 (2.5)	199 (2.7)	0.89 (0.66-1.21)
AIDS	3 (<0.01)	0	3 (<0.01)	NA
ESA medication				
Short acting	7590 (77.6)	1615 (69.6)	5975 (80.1)	1.76 (1.57-1.96)
Long acting	2186 (22.4)	705 (30.4)	1481 (19.9)	

Abbreviations: ESA, erythropoiesis-stimulating agent; NA, not applicable; OR, odds ratio.

^a^ Matched for birth year, sex, follow-up time, year of dialysis initiation, and dialysis modality.

### Association Between ESA Use and Cancer Development

The median of the mean weekly dose of each ESA drug was 10 325.5 U/week for epoetin alfa, 38.7 µg/week for darbepoetin alfa, and 35.0 µg/week for methoxy polyethylene glycol–epoetin beta. During follow-up, the case group had a higher proportion of patients who received high-dose ESAs (53.8%) than the control group (48.8%). The medians of the mean weekly doses of ESAs were 10 944.7 (case group) and 10 202.4 U/week (control group) for epoetin alfa, 38.6 (case group) and 38.7 µg/week (control group) for darbepoetin alfa, and 35.3 (case group) and 34.6 µg/week (control group) for methoxy polyethylene glycol–epoetin beta. In the conditional logistic regression model analysis without adjustment, high-dose ESA use was associated with greater odds of cancer development (crude OR, 1.22; 95% CI, 1.11-1.34) compared with low-dose use. In both model 1 (adjusted for demographics and health care facility) and model 2 (further adjusted for comorbidities), high-dose ESAs were associated with greater odds of cancer development (AOR, 1.23; 95% CI, 1.11-1.35) (Table 2). In an additional sensitivity analysis restricting ESA exposure to up to 6 months prior to the end of follow-up, 2009 cases were matched to 6537 controls. This sensitivity analysis showed that the odds of cancer development were significantly greater in patients who received high-dose ESAs than in low-dose users (AOR for model 2, 1.31; 95% CI, 1.18-1.46; *P* < .001) (eTable 2 in Supplement 1).

**Table 2.** Association Between ESA Use and Cancer Development

ESA measure	No. (%)		Crude OR (95% CI)	*P* value	Model 1^a^		Model 2^b^	
	Cases	Controls			AOR (95% CI)	*P* value	AOR (95% CI)	*P* value
Low dose	1072 (46.2)	3816 (51.2)	1 [Reference]	<.001	1 [Reference]	<.001	1 [Reference]	<.001
High dose	1248 (53.8)	3640 (48.8)	1.22 (1.11-1.34)		1.23 (1.11-1.35)		1.23 (1.11-1.35)	

Abbreviations: AOR, adjusted odds ratio; ESA, erythropoiesis-stimulating agent; OR, odds ratio.

^a^ In addition to the matching variables for the nested case-control study design (age, sex, follow-up time, year of dialysis initiation, and dialysis modality), model 1 was adjusted for demographic information (residential area and income level) and health care facilities.

^b^ Model 2 was further adjusted for comorbidities (diabetes, hypertension, dyslipidemia, myocardial infarction, heart failure, peripheral vascular disease, cerebrovascular disease, dementia, chronic pulmonary disease, connective tissue disease, peptic ulcer disease, liver disease, hemiplegia, and AIDS) in addition to the variables adjusted for in model 1.

During the study period, the 3 sites at which cancer was most frequently diagnosed were the digestive (n = 620 cases), respiratory (n = 270 cases), and kidney (n = 249 cases) systems. When assessed for each site-specific cancer, high-dose ESA use was associated with greater odds of cancer development in the digestive system (AOR, 1.37; 95% CI, 1.14-1.65), respiratory system (AOR, 1.48; 95% CI, 1.12-1.97), and ill-defined and unspecified sites (AOR, 1.64; 95% CI, 1.15-2.33), whereas no significant associations were found for other site-specific cancers (eTable 3 in Supplement 1).

### Subgroup Analyses

To test effect modification in the association between ESA use and cancer development, further analyses were performed according to prespecified subgroups. Overall, no significant interaction was observed across most subgroups, indicating that the greater odds of cancer development associated with high-dose ESA use were not significantly modified by the factors examined. However, interaction was observed among subgroups stratified by age. The AOR of high-dose ESA use for cancer development was 0.90 (95% CI, 0.77-1.05) among patients aged younger than 60 years, whereas it was 1.47 (95% CI, 1.30-1.67) among those aged 60 years or older (*P* < .001 for interaction) (Table 3).

**Table 3.** Subgroup Analyses

Subgroup	ESA dose	No. (%)		AOR (95% CI)^a^
		Case	Control	
Age, y				
<60	Low	449 (49.9)	1435 (47.9)	1 [Reference]
	High	450 (50.1)	1559 (52.1)	0.90 (0.77-1.05)
≥60	Low	623 (43.8)	2381 (53.4)	1 [Reference]
	High	798 (56.2)	2081 (46.6)	1.47 (1.30-1.67)
Sex				
Male	Low	727 (47.3)	2414 (50.7)	1 [Reference]
	High	809 (52.7)	2346 (49.3)	1.14 (1.01-1.29)
Female	Low	345 (44.0)	1402 (52.0)	1 [Reference]
	High	439 (56.0)	1294 (48.0)	1.34 (1.14-1.59)
Dialysis modality				
Hemodialysis	Low	993 (45.6)	3637 (51.1)	1 [Reference]
	High	1187 (54.5)	3483 (48.9)	1.23 (1.11-1.36)
Peritoneal dialysis	Low	79 (56.4)	179 (53.3)	1 [Reference]
	High	61 (43.6)	157 (46.7)	0.78 (0.50-1.22)
ESA medication				
Short acting	Low	731 (45.3)	3063 (51.3)	1 [Reference]
	High	884 (54.7)	2912 (48.7)	1.29 (1.14-1.45)
Long acting	Low	341 (48.4)	753 (50.8)	1 [Reference]
	High	364 (51.6)	728 (49.2)	1.09 (0.82-1.46)
Health care facility				
Primary care clinic	Low	974 (46.8)	3554 (52.0)	1 [Reference]
	High	1108 (53.3)	3286 (48.0)	1.26 (1.13-1.40)
Hospital	Low	98 (41.2)	262 (42.5)	1 [Reference]
	High	140 (58.8)	354 (57.5)	0.75 (0.34-1.65)
Diabetes				
Absence	Low	410 (45.7)	1402 (50.3)	1 [Reference]
	High	488 (54.3)	1383 (49.7)	1.27 (1.04-1.55)
Presence	Low	662 (46.6)	2414 (51.7)	1 [Reference]
	High	760 (53.4)	2257 (48.3)	1.16 (1.01-1.33)
Hypertension				
Absence	Low	40 (57.1)	121 (60.8)	1 [Reference]
	High	30 (42.9)	78 (39.2)	1.85 (0.69-4.97)
Presence	Low	1032 (45.9)	3695 (50.9)	1 [Reference]
	High	1218 (54.1)	3562 (49.1)	1.21 (1.09-1.34)

Abbreviations: AOR, adjusted odds ratio; ESA, erythropoiesis-stimulating agent.

^a^ Adjusted for age, sex, follow-up time, year of dialysis initiation, dialysis modality, residential area, income level, health care facility, and comorbidities such as diabetes, hypertension, dyslipidemia, myocardial infarction, heart failure, peripheral vascular disease, cerebrovascular disease, dementia, chronic pulmonary disease, connective tissue disease, peptic ulcer disease, liver disease, hemiplegia, and acquired immunodeficiency syndrome.

## Discussion

In this population-based retrospective nested case-control study, high-dose ESA use was associated with greater odds of cancer development among patients with kidney failure receiving long-term dialysis. This association was also found regardless of comorbidities such as diabetes and hypertension, dialysis modality, and type of ESA medication, and the odds were higher in older patients. Given that patients with kidney failure are typically underrepresented in long-term randomized clinical trials due to the high morbidity and complex clinical course of the disease, this clinical evidence provides important insights into the potential cancer risk associated with high-dose ESA use in this understudied population.

Earlier studies in oncology literature have demonstrated increased tumor progression with ESA use. In the ENHANCE trial, patients with head and neck cancer treated with epoetin beta had an increased risk of locoregional progression and death.^21^ Similarly, the BEST study of patients with metastatic breast cancer receiving chemotherapy showed that the 12-month survival rate was lower in the ESA-treated group than in the control group; this difference was attributed to disease progression and increased number of thrombotic and vascular events.^22^ Recognizing the relatively high ESA doses and hemoglobin targets in earlier trials (≥14 vs <12 g/dL in women and ≥15 vs <13 g/dL in men in ENHANCE study and 12 to 14 g/dL in BEST study), a subsequent analysis that comprehensively reviewed 6 meta-analyses encompassing 56 trials suggested no overall effect of ESAs on cancer progression.^23^ However, evidence of the association between ESAs and the risk of de novo cancer in patients with kidney disease remains scarce. To our knowledge, there has been 1 nested case-control study^19^ of 4574 Canadian patients undergoing dialysis that evaluated ESA exposure 6 to 9 months prior to cancer diagnosis. During the median follow-up of 1.8 years, ESA exposure was associated with overall greater odds of cancer, primarily driven by the high-dose group (weekly doses of >70 μg of darbepoetin alfa or equivalent epoetin alfa), compared with those unexposed. In contrast, the present study evaluated the mean weekly dose of ESAs received by each patient during an overall follow-up period of more than 6 years, thereby providing a more comprehensive assessment of cumulative exposure and long-term cancer development. Our findings on the association between high-dose ESA use and greater odds of cancer development in patients with kidney failure undergoing long-term dialysis align with several previous study findings of adverse cancer outcomes associated with ESA use. This association was found regardless of ESA type (short vs long acting) and even when ESA use was restricted to 6 months before cancer diagnosis to further minimize protopathic bias in sensitivity analysis. Meanwhile, considering the insufficient number of site-specific cancer outcomes in the present study, it is difficult to determine the association between ESA use and each specific cancer type. Further studies are therefore required to elucidate the pathologic mechanisms underlying ESA-mediated tumor progression in specific tumor types.

Notably, the AORs for high-dose ESA use and cancer development appeared to be higher in older patients. Several potential mechanisms could be postulated to explain this finding. ESAs can activate erythropoietin receptors not only in erythroid cells but also in other cell types, resulting in direct stimulation of malignant or premalignant cells to proliferate and resist apoptosis,^24^ or they may engage erythropoietin receptors on immune cells such as macrophages to create an immunosuppressive microenvironment that promotes tumor growth.^25,26^ Among older individuals experiencing immunosenescence, which can result in inadequate regulation of pro-oncogenic activity, advanced kidney disease requiring dialysis may also contribute to the development of a chronic inflammatory milieu,^27^ further exacerbating cancer susceptibility. Although reports regarding the influence of ESAs on tumor progression are conflicting, the findings of this study suggest that ESAs alone may not have direct impacts in cancer outcomes. Rather, the synergy between high-dose ESA exposure and the altered biology of older patients with kidney disease, including mutational burden, diminished immune surveillance, and chronic inflammation, provides a plausible explanation for the age-specific association with cancer development.

### Limitations

This study has several limitations. First, the possibility of residual bias due to potential unmeasured confounders inherent to observational studies cannot be excluded. In particular, insufficient information precluded consideration of lifestyle-related risk factors for cancer development, including tobacco use and alcohol consumption, as potential confounders. Information about functional iron deficiency induced by malignant neoplasms, specifically transferrin saturation and ferritin levels, was also unavailable in the NHIS database and could not be included in the analysis. Furthermore, the choice of ESA or the health care facility where dialysis was performed might have been influenced by the patient’s clinical status or systemic condition or the treating clinician’s preference. Nevertheless, the present study used a nested case-control design to minimize selection bias, which is often more pronounced in conventional case-control studies based on observational data that select cases and controls within the same source population.^28^ Moreover, analyses were further adjusted not only for clinical factors at the patient level but also for sociodemographic characteristics and health care facility type, thereby accounting for variation in clinical practice patterns across facilities. However, this statistical method is insufficient to determine causality between exposure and outcome. Specifically, it remains possible that the need for high ESA doses reflected an early undiagnosed manifestation of cancer rather than that the high ESA doses were a causal factor. However, because the sensitivity analysis considering ESA exposure only up to 6 months before the end of follow-up yielded results consistent with those of the main analysis, it is difficult to conclude that the findings were completely negated. Second, hemoglobin levels, which may be related to cancer development, could not be obtained from the NHIS database, making it impossible to directly evaluate the appropriateness of ESA use based on hemoglobin levels. Excessive elevation of hemoglobin levels following ESA administration has been demonstrated to increase cancer risk.^21,22^ Nevertheless, this intrinsic limitation is partially mitigated by clinical settings in Korea. Both the Korean NHIS and the Health Insurance Review and Assessment Service require regular monthly checkups at dialysis facilities to ensure appropriate dialysis-related treatment and medication use. They also limit insurance coverage for ESA treatment if the measured hemoglobin level exceeds 11.0 g/dL, and this target level remained constant throughout the study period. Consequently, clinicians are obligated to administer ESAs at sufficient dosages to elevate hemoglobin levels within this limit. The concern regarding ESA overuse has already been reported to be low among patients with kidney failure in Korea.^29^ Third, the administration route (intravenous vs subcutaneous) could not be differentiated. Although doses may differ slightly by administration route, this nondifferential misclassification error would be unlikely to affect the interpretation of the results. In Korea, intravenous administration is generally preferred during hemodialysis because of its convenience and associated procedural fees; thus, most patients undergoing hemodialysis receive ESAs intravenously rather than subcutaneously. Finally, the study population consisted exclusively of Korean participants, which may limit the generalizability of the findings to patients with kidney failure in other populations.

## Conclusions

In this case-control study, in patients with kidney failure undergoing long-term dialysis, high-dose ESA use was associated with greater odds of cancer development, with the odds being higher in older patients. Therefore, caution should be exercised to avoid aiming for excessively high hemoglobin levels during ESA therapy.

## Notes

Supplement 1. **eTable 1.** Diagnostic or Procedure Codes Used to Define Prevalent Comorbid Conditions and Outcomes **eTable 2.** Association Between the Use of ESAs Up to 6 Months Prior to the End of Follow-up and Cancer Development **eTable 3.** Association Between the Use of ESAs and the Development of Common Site-Specific Cancers **eFigure.** Covariate Balance Measured by Standardized Mean Differences

Supplement 2.
Data Sharing Statement
