# Wording Change in Introduction

**DOI:** 10.1001/jamanetworkopen.2026.9379

**Published:** 2026-04-02

In the Original Investigation titled “Erythropoiesis-Stimulating Agents and Development of Cancer Among Patients Receiving Dialysis,”^1^ published February 27, 2026, a wording change is required for the first sentence in the second paragraph of the Introduction citing to Elliot and Sinclair,^2^ Diskin et al,^3^ and Cao^4^ (references 8-10 in the published article). This change emphasizes the low quality of existing evidence and lack of proven causal relationship between erythropoiesis-stimulating agents (ESAs) and tumor progression noted in the Elliot and Sinclair^2^ study. The revised material reads as follows: “Mechanistic studies conducted to identify the underlying causes of the disadvantages associated with ESA use have revealed that ESAs can induce the activation of erythropoietin receptors, although the clinical evidence for increased angiogenesis and tumor growth remains controversial and subject to debate due to low-quality evidence in existing literature.^8-10^ Furthermore, large-scale evidence of cancer outcomes among patients with kidney failure remains sparse and inconsistent.” This article has been corrected.^1^
