# Supplementary material for: Erythropoiesis-Stimulating Agents and Development of Cancer Among Patients Receiving Dialysis
Source: JAMA Netw Open. 2026 Feb 27;9(2):e260140. doi: 10.1001/jamanetworkopen.2026.0140 (PMC12949436; doi:10.1001/jamanetworkopen.2026.0140)
Supplement: Supplement 1. — eTable 1. Diagnostic or Procedure Codes Used to Define Prevalent Comorbid Conditions and Outcomes eTable 2. Association Between the Use of ESAs Up to 6 Months Prior to the End of Follow-up and Cancer Development eTable 3. Association Between the Use of ESAs and the Development of Common Site-Specific Cancers eFigure. Covariate Balance Measured by Standardized Mean Differences [file jamanetwopen-e260140-s001.pdf]

Supplemental Online Content

Kim JY, JK Lee, TI Chang, HW Kim. Erythropoiesis-stimulating agents and development of cancer among patients receiving dialysis. *JAMA Netw Open*. 2026;9(2):e260140. doi:10.1001/jamanetworkopen.2026.0140

eTable 1. Diagnostic or Procedure Codes Used to Define Prevalent Comorbid Conditions and Outcomes

eTable 2. Association Between the Use of ESAs Up to 6 Months Prior to the End of Follow-up and Cancer Development

eTable 3. Association Between the Use of ESAs and the Development of Common Site-Specific Cancers

eFigure. Covariate Balance Measured by Standardized Mean Differences

This supplemental material has been provided by the authors to give readers additional information about their work.

© 2026 Kim JY et al. *JAMA Network Open*.

eTable 1. Diagnostic or procedure codes used to define prevalent comorbid conditions and outcomes.

Conditions	ICD-10 or procedure codes
**Comorbidities**	
Diabetes	E10–14
Hypertension	I10–13, I15
Dyslipidemia	E78.0–78.5
Myocardial infarction	I21, I25.2
Heart failure	I50
Peripheral vascular disease	I702, I73
Cerebrovascular disease	I60-64, I69
Dementia	F00, F01, F03, G30
Chronic pulmonary disease	J42–J47, J60–67, J70.1, J70.3
Connective tissue disease	M05, M06, M30-36, M45
Peptic ulcer disease	K25–28
Liver disease	B18, I85, I86.4, I98.2, K70.3, K70.4, K71.1, K71.3, K71.4, K71.5, K71.7, K72.1, K72.9, K73, K74.3–6, Z94.4
Hemiplegia	G04.1, G11.4, G80.1, G81, G82, G83.0–4, G83.9
Acquired immunodeficiency syndrome	B20–22, B24
**Outcomes**	
Oral cavity and pharynx	C00–C14
Digestive	C15–C21, C23–C26
Liver and intrahepatic bile ducts	C22
Respiratory	C30–C39
Bone and cartilage	C40–C41
Melanoma	C43
Soft tissue/connective tissue	C45–C49
Breast	C50
Female genital organs	C51–C58
Male genital	C60, C62–C63
Prostate	C61
Kidney	C64
Urinary tract	C65–C68
Central nervous system	C69–C72
Endocrine	C73–C75
Unknown origin	C76–C80
Multiple myeloma	C90
Hematological	C81–C89, C91-C96
Multiple primary sites	C97–C98

Note: To identify participants' previous medical history, *the International Classification of Disease–10th Revision* (ICD-10) coding of covered inpatient and outpatient visits from medical claim records were used.

© 2026 Kim JY et al. *JAMA Network Open*.

eTable 2. Association between the use of ESA up to 6 months prior to the end of follow-up and cancer development.

	Cases (%) (n= 2,009)	Controls (%) (n= 6,537)	Crude		Model 1		Model 2	
			OR (95% CI)	P value	AOR (95% CI)	P value	AOR (95% CI)	P value
Low dose	847 (42.2)	3,236 (49.5)	Reference	<0.001	Reference	<0.001	Reference	<0.001
High dose	1,162 (57.8)	3,301 (50.5)	1.34 (1.21-1.48)		1.32 (1.19-1.47)		1.31 (1.18-1.46)	

In addition to the matching variables for the nested case-control study design (age, sex, follow-up time, year of dialysis initiation, and dialysis modality), Model 1 was adjusted for demographic information (residential area and income level) and healthcare facility. Model 2 was further adjusted for comorbidities (diabetes, hypertension, dyslipidemia, myocardial infarction, heart failure, peripheral vascular disease, cerebrovascular disease, dementia, chronic pulmonary disease, connective tissue disease, peptic ulcer disease, liver disease, hemiplegia, acquired immunodeficiency syndrome), in addition to variables adjusted in Model 1.

Abbreviation: AOR, adjusted odds ratio; CI, confidence interval; ESA, erythropoiesis-stimulating agents.

© 2026 Kim JY et al. *JAMA Network Open*.

eTable 3. Association between the use of ESA and the development of common site-specific cancers.

	Case (%)	Control (%)	AOR (95% CI)	P value
	Digestive system (n=620)			
Low dose	278 (44.8)	1,257 (51.7)	Reference	0.001
High dose	342 (55.2)	1,173 (48.3)	1.37 (1.14-1.65)	
	Liver and intrahepatic bile ducts (n=189)			
Low dose	95 (50.3)	386 (52.0)	Reference	0.40
High dose	94 (49.7)	357 (48.1)	1.19 (0.79-1.81)	
	Respiratory system (n=270)			
Low dose	117 (43.3)	561 (52.7)	Reference	0.01
High dose	153 (56.7)	504 (47.3)	1.48 (1.12-1.97)	
	Breast (female) (n=113)			
Low dose	61 (54.0)	231 (52.4)	Reference	0.91
High dose	52 (46.0)	210 (47.6)	1.03 (0.65-1.62)	
	Female genital organs (n=55)			
Low dose	28 (50.9)	118 (54.4)	Reference	0.72
High dose	27 (49.1)	99 (45.6)	1.14 (0.56-2.30)	
	Prostate (n=72)			
Low dose	33 (45.8)	159 (56.2)	Reference	0.31
High dose	39 (54.2)	124 (43.8)	1.40 (0.73-2.68)	
	Kidney (n=249)			
Low dose	125 (50.2)	465 (48.5)	Reference	0.36
High dose	124 (49.8)	494 (51.5)	0.87 (0.65-1.17)	
	Urinary tract (n=88)			
Low dose	42 (47.7)	174 (50.3)	Reference	0.62
High dose	46 (52.3)	172 (49.7)	1.14 (0.67-1.94)	
	Endocrine (n=92)			
Low dose	47 (51.1)	160 (45.6)	Reference	0.58
High dose	45 (48.9)	191 (54.4)	0.87 (0.52-1.44)	
	Hematologic except myeloma (n=50)			
Low dose	22 (44.0)	95 (48.5)	Reference	0.86
High dose	28 (56.0)	101 (51.5)	0.94 (0.45-1.94)	
	Ill-defined and unspecified (n=184)			
Low dose	77 (41.9)	372 (51.5)	Reference	0.006
High dose	107 (58.1)	351 (48.5)	1.64 (1.15-2.33)	
	Multiple sites (n=156)			
Low dose	69 (44.2)	314 (51.1)	Reference	0.10
High dose	87 (55.8)	301 (48.9)	1.39 (0.94-2.07)	

In addition to the variables used for matching (age, sex, follow-up time, year of dialysis initiation, and dialysis modality), adjusted for residential area, income level, healthcare facility, and comorbidities of diabetes, hypertension, dyslipidemia, myocardial infarction, heart failure, peripheral vascular disease, cerebrovascular disease, dementia, chronic pulmonary disease, connective tissue disease, peptic ulcer disease, liver disease, hemiplegia, acquired immunodeficiency syndrome.

Abbreviation: AOR, adjusted odds ratio; CI, confidence interval; ESA, erythropoiesis-stimulating agents.

© 2026 Kim JY et al. *JAMA Network Open*.

eFigure. Covariate balance measured by standardized mean differences.

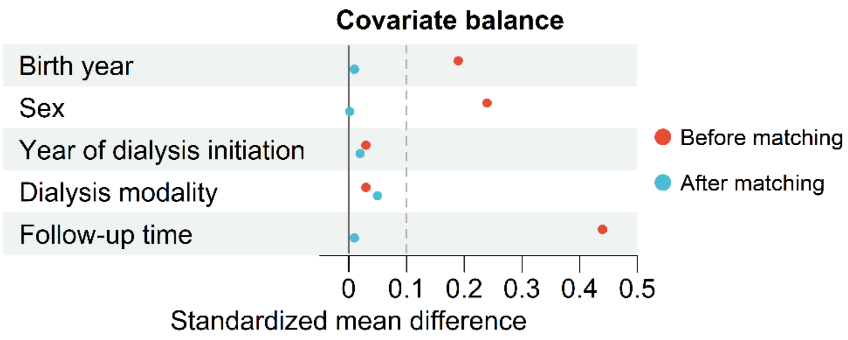

© 2026 Kim JY et al. *JAMA Network Open*.
