# Supplementary material for: Erythropoiesis-Stimulating Agents and Development of Cancer Among Patients Receiving Dialysis
Source: JAMA Netw Open. 2026 Feb 27;9(2):e260140. doi: 10.1001/jamanetworkopen.2026.0140 (PMC12949436; doi:10.1001/jamanetworkopen.2026.0140)
Supplement: Supplement 2. — Data Sharing Statement [file jamanetwopen-e260140-s002.pdf]

Data Sharing Statement

Kim. Erythropoiesis-Stimulating Agents and Development of Cancer Among Patients Receiving Dialysis. *JAMA Netw Open*. Published February 27, 2026.
doi:10.1001/jamanetworkopen.2026.0140

Data
Data available: No

Additional Information
Explanation for why data not available: All relevant data are within the manuscript and supplemental materials. All data are available through approval and oversight by the Korean National Health Insurance Service at the National Health Insurance Data Sharing Service (https://nhiss.nhis.or.kr/bd/ab/bdaba000eng.do).
